# POU5F1 Enhances the Invasiveness of Cancer Stem-Like Cells in Lung Adenocarcinoma by Upregulation of MMP-2 Expression

**DOI:** 10.1371/journal.pone.0083373

**Published:** 2013-12-30

**Authors:** Yan-hong Xin, Bai-shi-jiao Bian, Xiao-jun Yang, Wei Cui, Hong-juan Cui, You-hong Cui, Xia Zhang, Chuan Xu, Xiu-wu Bian

**Affiliations:** 1 Institute of Pathology and Southwest Cancer Center, Southwest Hospital, Third Military Medical University, and Key Laboratory of Tumor Immunopathology, Ministry of Education of China, Chongqing, China; 2 Department of Oncology, General Hospital of PLA Chengdu Military Area Command, Chengdu, China; 3 State Key Laboratory of Silkworm Genome Biology, Institute of Sericulture and Systems Biology, Southwest University, Chongqing, China; 4 Yongchuan Hospital, Chongqing Medical University, Chongqing, China; Università degli Studi di Firenze, Italy

## Abstract

Lung cancer is the leading cause of cancer-related human deaths. Exploration of the mechanisms underlying the metastasis of cancer stem-like cells (CSLCs) will open new avenues in lung cancer diagnosis and therapy. Here, we demonstrated that CSLCs-derived from lung adenocarcinoma (LAC) cells displayed highly invasive and migratory capabilities via expressing high levels of POU5F1 and MMP-2. We found that POU5F1 directly regulated MMP-2 transcription via interaction with the promoter of MMP-2. POU5F1 knockdown in LACSLCs reduced MMP-2 protein abundance, leading to inhibition of the cell invasion, migration and tumorigenesis potentials of LAC cells. Clinically, aberrantly high expressions of POU5F1 and MMP-2 were inversely correlated with the survival of LAC patients, and the double-positive POU5F1 and MMP-2 showed the worst prediction for the patient’s poor survival. These results indicate that POU5F1 can bind to the MMP-2 promoter for the degradation of surrounding extracellular matrix, and therefore promote invasive and migratory capabilities of LACSLCs. Moreover, our data implicate that the pathological detection of the double-positive expressions for POU5F1 and MMP-2 will be useful as diagnostic and prognostic biomarkers in LAC to advance anti-metastasis therapy.

## Introduction

Lung cancer is the leading causes of human deaths worldwide [1]. Despite the great advances in lung cancer therapeutics, the metastasis and treatment failure often result in recurrence and high mortality. One possible reason underlying therapeutic failure and human deaths is the presence of residual malignant cells that ultimately give rise to secondary tumors and metastases [2].

Up to date, accumulating evidence has been emerging that a small population of cells possess tumor-initiating activity in lung cancer and are termed as ‘‘cancer stem-like cells (CSLCs)’’ [3], [4]. The presence of a small subpopulation of CSLCs might explain why so many lung cancers recur after treatment with surgery, chemotherapy and radiotherapy, even when most of the malignant cells seem to be killed after treatment [5], [6]. Therefore, it is critical to uncover the biological characteristics and molecular mechanisms for the initiation and progression of CSLCs for the development of novel therapies. Previously, we successfully established an *in vitro* sphere culture system to isolate and enrich “lung adenocarcinoma cancer stem-like cells” (LACSLCs) and revealed that the POU homeobox gene family transcription factor POU5F1 (also known as Oct4) acts as a key regulator for the retention of self-renewal and tumorigenicity of LACSLCs [7].

POU5F1 is required to maintain the self-renewal of embryonic stem (ES) cells, and contributes to the stemness, tumorigenesis and metastasis of CSLCs [8]–[10]. It has been recently reported that POU5F1 promotes invasion and metastasis of some solid tumors through enhanced degradation of surrounding extracellular matrix, suggesting that this transcription factor may be useful as a potential therapeutic target against cancer and a novel tumor biological and prognostic marker [11], [12]. However, the mechanisms of aberrant POU5F1 expression in lung cancer underlying invasion and metastasis remain elusive.

In the present study, we demonstrated that POU5F1 expression was associated with the invasion and migration of LACSLCs and explored the underlying molecular mechanisms. The clinical significance of POU5F1 and matrix metalloproteinase 2 (MMP-2) expressions in the patients with lung adenocarcinoma (LAC) was also investigated.

## Materials and Methods

### Ethics statement

This study was strictly carried out in accordance with the recommendations in the Guide for the Care and Use of Laboratory Animals approved by the Institutional Animal Care and Use Committee of the Third Military Medical University. Human primary LAC specimens were harvested with patient consent. Each participant in this study was informed with written consent. Human primary LAC specimens were harvested and the protocol was approved by the Medical Ethics Committee of Southwest Hospital of the Third Military Medical University. Animal experimental protocols were performed in accordance with National Institutes of Health Sciences and approved by the Institutional Animal Care and Use Committee of the Third Military Medical University.

### Cell culture

Human non-small-cell lung cancer (NSCLC) cell line A549 was purchased from the American Type Culture Collection (ATCC) (Rockville, MD) and cultured in DMEM medium supplemented with 10% fetal bovine serum (FBS), 100 U/ml penicillin and 100 U/ml streptomycin (Gibco, USA) in a humidified 37 °C incubator with 5% CO_2_ atmosphere. Tumor sphere culture was performed as described previously [4], [13]–[15].

### Lentiviral shRNA, siRNA construction and transfection

Lentiviral constructs and the primers targeting human Pou5f1 short hairpin sequences were designed and prepared by Sunbio Medical Biotechnology (Shanghai, China). The shRNA expression lentivirus vectors were prepared by transient transfection of the lentiviral constructs into 293T cells to produce viruses. The resulting lentiviral POU5F1-shRNA and control-shRNA were used to infect A549 cells. A control-shRNA was designed by mutation of the antisense nucleotides in the POU5F1 shRNA sequence. The plasmid constructs carrying a siRNA sequence targeting MMP-2 were designed and constructed as previously described [16]. Transfections were performed with Lipofectamine 2000 (Invitrogen, Carlsbad, CA) according to the manufacturer’s instructions.

### *In vitro* wound healing assay

Confluent cells were wounded using a 200-µL pipette tip in six-well culture plates and incubated in DMEM with 5% or 10% of fetal bovine serum in the presence or absence of mitomycin C (10 µg/mL). The wound width was photographed at 0h, 16h and 24h post-scratch under a phase-contrast microscope. The migration distances were measured and quantified as described previously [12]. The computer-assisted image software was used to quantify cell migration relative to the grid line border.

### *In vitro* invasion assay

The cell invasive capacity was determined in a 24-well format using Transwell® inserts with 8 µm pore size, coated with Matrigel® (BD Biosciences, USA) as described previously [17]. Tumor spheres were trypsinized, resuspended in serum-free DMEM medium, and placed in the upper chambers of the Transwell® inserts (5×10^4^/well), and DMEM medium containing 10% FBS was added to the lower chambers. The cells were allowed to invade through the membrane for 24 h and subsequently fixed with 4% paraformaldehyde in PBS. The non-invasive cells in the upper chambers were wiped off by a cotton swab and invasive cells were stained with crystal violet solution. The numbers of cells that had invaded to the lower surface of the inserts were counted under a light microscope.

### Tumorigenicity analysis *in vivo*

For mice xenografts, adherent A549 monolayer cells and tumor spheres were dissociated to obtain single-cell suspensions. An equal number of cells (5×10^5^) were diluted in serum-free DMEM medium and injected subcutaneously to four-week-old male nude mice (*n* = 5 each, Center of Experimental Animals, Third Military Medical University, China). Mice were monitored every week for the appearance of subcutaneous tumors. At the end of 7 weeks, all mice were sacrificed and tumor engrafts were removed and measured. Tumor volume (TV) was calculated using the following formula: TV (mm^3^)  =  *d* ^2^×*D*/2, where d and D represent the shortest and the longest diameters, respectively. In addition, the tumor tissues were fixed in buffered formalin and subsequently performed to tumor histology and immunohistochemical analysis.

### Quantitative Real-time PCR (qRT-PCR)

Total RNA was extracted from cancer cells using Trizol Reagent (Invitrogen, USA). qRT-PCR was performed using SYBR PrimeScript RT-PCR kit (TaKaRa, Japan) on a Rotor-Gene 6000 real-time genetic analyzer (Corbett Life Science, USA). The primers used for the PCR amplification of internal fragments of MMP-2 are listed as follows: 5′-CCACTGCCTTCGATACAC-3′ (sense); 5′-GAGCCACTCTCTGGAATCTTAAA-3′(anti-sense). Glyceraldehyde-3-phosphate dehydrogenase (GAPDH) was amplified as an internal control. Each sample was tested at least in triplicates.

### Western blot analysis

Cell lysates of A549 monolayer cells and tumor spheres were prepared using RIPA buffer with protease inhibitors and quantified using BCA protein assay (Pierce, Rockford, IL). Protein samples (20 µg) were loaded onto a 10% SDS-PAGE and transferred onto the PVDF membranes (Millipore, USA). Immune complexes were formed by incubation of mouse monoclonal MMP antibody (1∶400; Abcam, USA), rabbit polyclonal POU5F1 antibody (1∶500; BD Biosciences, USA) and rabbit polyclonal Actin antibody (1∶5000; Abcam, USA) at 4°C overnight, followed by incubation with horseradish peroxidase–conjugated secondary antibodies to mouse or rabbit IgG (1∶5000; Invitrogen, USA). Immunoreactive protein was visualized using SuperSignal West Femto Trial Kit (Pierce, Rockford, IL) by an enhanced chemiluminescence detection system.

### Chromatin immunoprecipitation (ChIP)

ChIP assay was performed with a ChIP-IT kit according to the manufacturer’s protocol. Ultrasonic shearing conditions resulted in relatively uniformed DNA fragment in the size of ∼300 bp. The remaining procedures were completed as previously described [7]. Genomic DNA was extracted from the precipitates and amplified by qRT-PCR. The primer pairs used for PCR to amplify MMP-2 promoter region containing POU5F1 binding element were: 5′- CATGACAACAGGCTTTGGATTAG-3′ (sense) and 5′- AACAAACTCTTAGGCAACGAACC -3′ (anti-sense).

### Immunohistochemistry (IHC)

Immunohistochemical staining of tumor tissues and xenografts was performed on specimens using streptavidin-biotin peroxidase complex (SABC) method. Briefly, xenograft samples were fixed in 4% paraformaldehyde at 4°C for 72 h and embedded in paraffin. The paraffin sections were incubated with primary antibodies at 4°C overnight. The slides were then reacted with biotinylated goat anti-mouse/rabbit IgG and avidin-biotin complex (ABC) (Beyotime, China). For visualization of the antibody-antigen complex, chromogen reaction was carried out with diaminobenzidine (DAB) and the slides were examined under a light microscope. For quantification, IPP software (image-pro plus 6.0) was used to analyze the optical density of the images within 5 random fields at 400× magnification. The average optical density (AOD), namely IOD/area, was calculated.

### Transfection and luciferase reporter assay

Construction and transient transfection of firefly luciferase reporter plasmid were performed as previously described [18]. The sequence of pGL3-MMP-2 promoter construct was confirmed (Sunbio Medical Biotechnology, China). The shRNA-Control and shRNA-Pou5f1-infected A549 LACSLCs were seeded into 24-well plates (1×10^5^ in each well). After 24 hours, the cells were co-transfected with pGL3-MMP-2 promoter and Renilla vector (pRL-TK: Promega, USA). Cell lysates were collected at 48h post-transfection and the relative luciferase reporter enzyme activity was measured using the Dual-Luciferase® Reporter Assay System (Promega, USA). Firefly luciferase activity was normalized to Renilla luciferase activity for each sample.

### Tissue microarray (TMA) analysis

A total of 55 patients with primary LAC who received surgical resection were included. Tumor grades were defined according to the criteria of the World Health Organization. The Tumor-Node-Metastasis (pTNM) status of all LACs was assessed according to the criteria of the sixth edition of the TNM classification of the International Union Against Cancer [19]. The clinico-pathological characteristics of the patients were summarized in Table 1. The TMA was constructed as previously described [20].

**Table 1:** The expression of POU5F1 and MMP-2 in 55 cases of lung adenocarcinoma.

Characteristics	Subcategory	Cases	POU5F1high expression	*P* value	MMP-2high expression	*P* value
Age (years) a	≤ 59	21	10(47.6%)	0.968	16 (76.2%)	0.498
	> 59	34	16(47.1%)		23 (67.6%)	
Gender	Male	30	15 (50.0%)	0.657	20 (66.7%)	0.448
	Female	25	11 (44.0%)		19 (76.0%)	
Tumor grade	G1	6	1 (16.7%)	0.280	5 (83.3%)	0.664
	G2	20	10 (50.0%)		13 (14.2%)	
	G3	29	15 (51.7%)		21 (72.4%)	
pT status	pT1-2	42	19 (45.2%)	0.587	31 (73.8%)	0.395
	pT3-4	13	7 (53.8%)		8 (61.5%)	
pN status	pN0	27	9 (33.3%)	0.042	19 (70.4%)	0.931
	pN1-3	28	17 (60.7%)		20 (71.4%)	
M status	M0	53	24 (45.3%)	0.128	38(71.7%)	0.507
	M1	2	2 (100.0%)		1(50.0%)	
Stage	I	24	7 (29.2%)	0.074	18 (75.0%)	0.872
	II	9	5 (55.6%)		6 (66.7%)	
	III	20	12 (60.0%)		14 (70.0%)	
	IV	2	2 (100.0%)		1 (50.0%)	

^a^ Mean age.

### Statistical analysis

All experiments were performed at least three times in triplicates. Data were presented as the mean ± SEM. Statistical analysis was performed using SPSS13.0 software. Statistically significant difference was assessed by one-way analysis of variance (ANOVA) followed by multiple mean comparisons by Student–Newman–Keul’s test. Statistical difference was considered significant if *P*-value was less than 0.05.

## Results

### LACSLCs display highly migratory and invasive abilities

**Figure 1:**
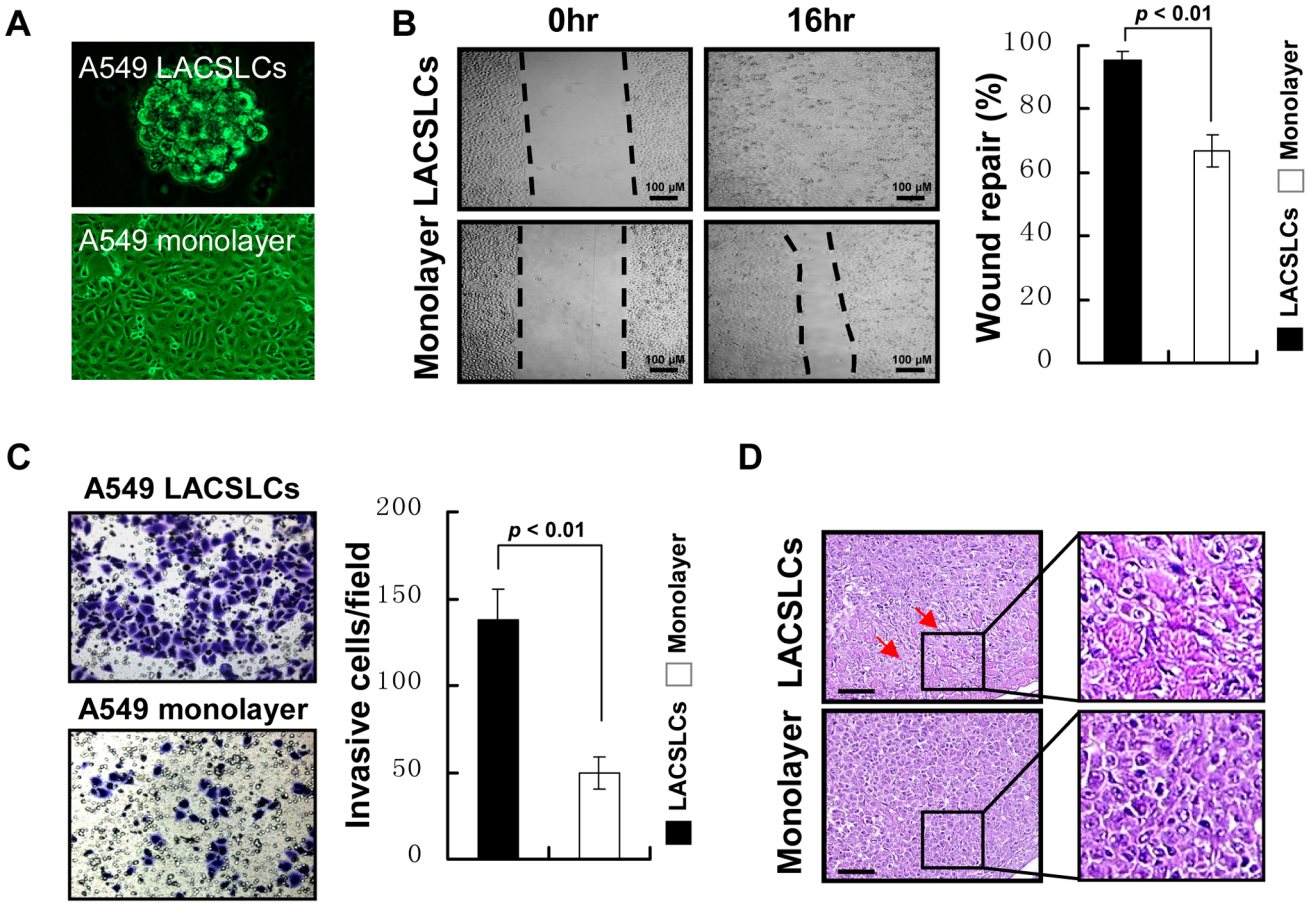
LACSLCs derived from A549 cells exhibit highly invasive and migratory capabilities. (**A**) Morphology of sphere-LACSLCs derived from lung cancer A549 cell line. Representative images from light microscopy (monolayer cells 10×, LACSLCs 20×) are shown. (B) Wound healing assay for cell migration of A549 LACSLCs and A549 monolayer cells. Representative images of cell migration into the wounded area at 0 and 16 hours post-injury (*left panel*) are shown. Quantitative analysis shows wound repair capability of migrating cells at 16 h post-injury (*right panel*). (**C**) Comparison of invasive capability in A549 LACSLCs and monolayer cells detected by transwell assay. Representative images of invading cells visualized by crystal violet staining (20×) (*left panel*). Quantitative analysis of cell invading capacity at 24 h after seeding (*right panel*). (**D**) Histological images by H&E staining under light microscopy. Tumors formed by A549 LACSLCs were highly invasive and the tumor cells invaded neighboring muscle layer (red arrows; 20×). All experiments were carried out at least in triplicates and the data are presented as the mean ± SEM. Student *t* test was performed to evaluate the difference.

In our study, we established a stable sphere culture system for isolating and enriching LACSLCs to elucidate their biological behaviors. As shown in Figure 1, A549 cells that were cultured in the SFM with EGF and bFGF generated non-adherent, multi-cellular sphere LACSLCs (Figure 1A), and these sphere LACSLCs displayed highly invasive and migratory capabilities *in vitro* (Figure 1B and C) and *in vivo* (Figure 1D). The results of *in vitro* wound-healing assay demonstrated that sphere LACSLCs migrated into the scratched area more rapidly than A549 monolayer cells. Furthermore, results from an *in vitro* Matrigel invasion assay showed that A549 LACSLCs displayed higher invasive capacity than A549 monolayer cells (137.5±17.7 versus 49.7±9.2 invading cells/field). Moreover, A549 LACSLCs exhibited higher tumorigenicity *in vivo* that led to larger tumor xenografts with enhanced invasion activity (Figure 1D). Overall, these results indicate that LACSLCs are highly migratory and invasive *in vitro* and *in vivo*.

### POU5F1 and MMP-2 are aberrantly high expressed in LACSLCs

**Figure 2:**
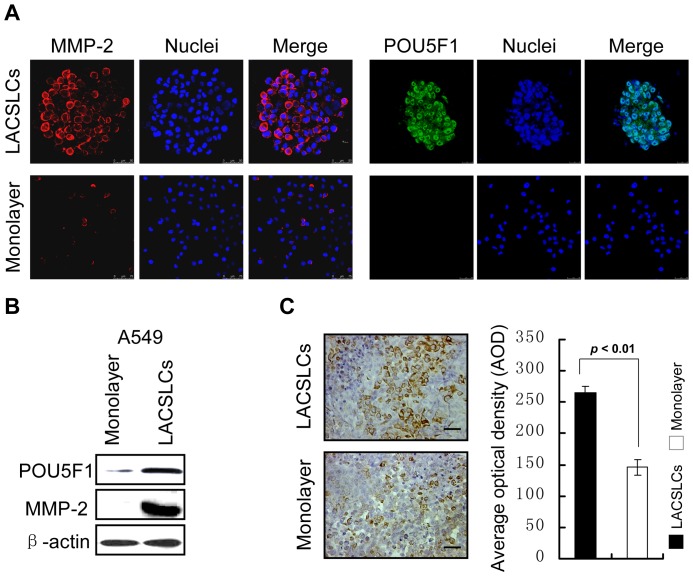
Examination of POU5F1 and MMP-2 by immunostaining and Western blot analyses. (**A**) Immunostaining of POU5F1 and MMP-2 observed by confocal scanning microscopy. (**B**) Expression of stem cell transcription factor POU5F1 was detected by Western blot. (**C**) Immunohistochemical analysis of MMP-2 protein in tumor tissues formed by A549 LACSLCs and monolayer cells. Representative images of MMP-2 expression by IHC staining (20×) (*left panel*). Quantitative analysis of MMP-2 protein levels in tumor tissues formed by A549 LACSLCs and monolayer cells. All experiments were carried out at least in triplicates and the data are presented as the mean ± SEM. Student *t* test was performed to evaluate the difference.

In order to elucidate the underlying mechanism for the high migratory and invasive capabilities of LACSLCs, we examined the stem cell transcription factor POU5F1 and the zinc protease for degradation of extracellular matrix MMP-2. Immunostaining (Figure 2A) and western blot (Figure 2B) analyses showed that MMP-2 and POU5F1 were aberrantly high expressed in A549 LACSLCs. Moreover, immunohistochemical analysis demonstrated that the expression level of MMP-2 in tumor tissues formed by A549 LACSLCs was higher than that of tumors formed by A549 monolayer cells (Figure 2C). Collectively, these results suggest that the aberrantly high expressions of POU5F1 and MMP-2 may play important roles in the regulation of cell migratory and invasive capabilities of LACSLCs.

### POU5F1 knockdown disrupts the cell invasion, migration and tumorigenesis potentials of LACSLCs

**Figure 3:**
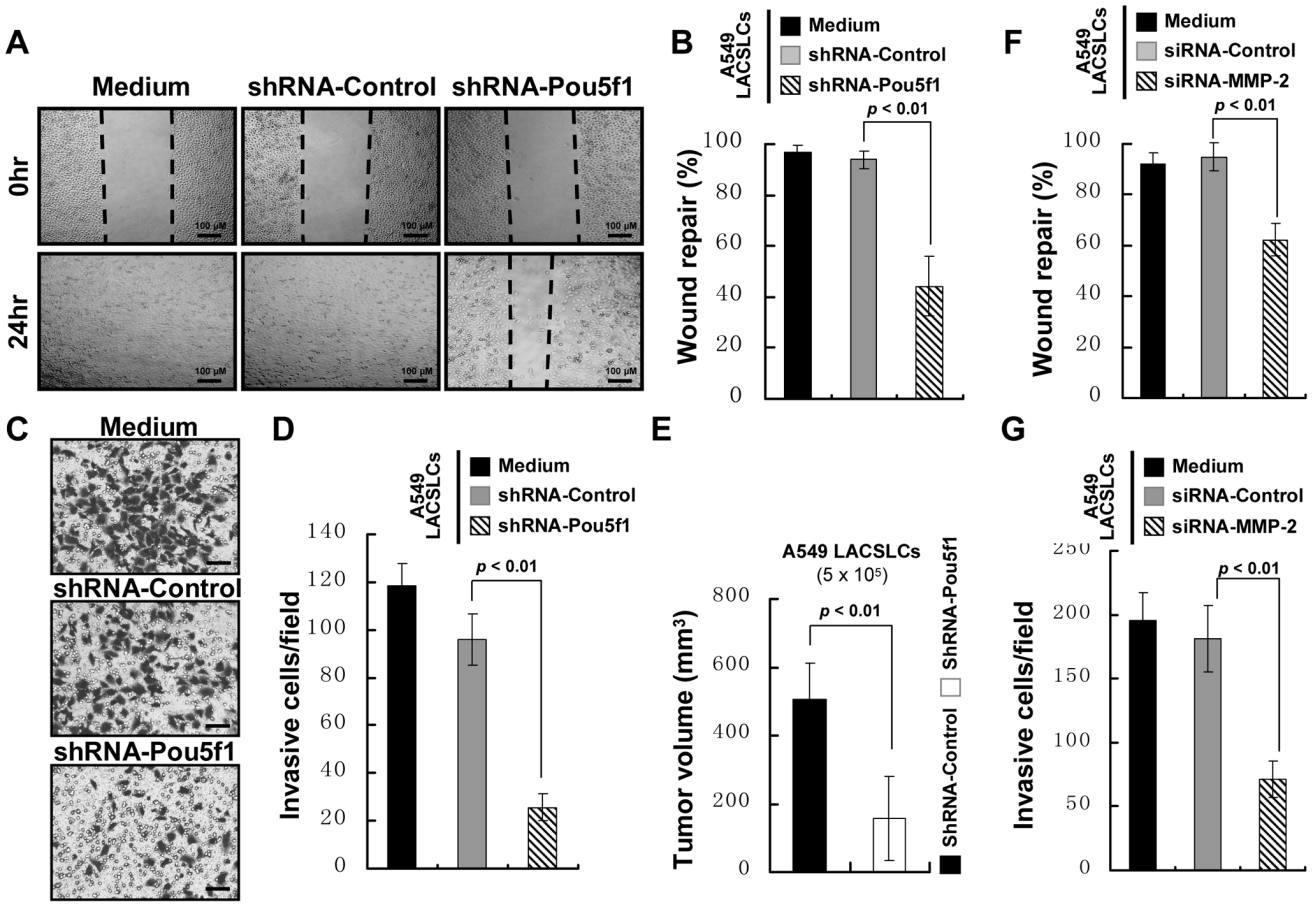
POU5F1 knockdown inhibits the cell invasion, migration and tumorigenesis potentials of A549 LACSLCs. (**A**) Representative images of wound healing assay for cell migration of shRNA-control or shRNA-Pou5f1-introduced A549 LACSLCs. (**B**) Quantitative analysis of wound repair capability of A549 LACSLCs containing shRNA-control or shRNA-Pou5f1 at 24 h after seeding. (**C**) Representative images of invading cells containing shRNA-control or shRNA-Pou5f1 visualized by crystal violet staining (20×). (**D**) Quantitative analysis of invasive ability of A549 LACSLCs containing shRNA-control or shRNA-Pou5f1. (**E**) Quantitative analysis of xenograft tumor volume formed by A549 LACSLCs containing shRNA-Control or shRNA-Pou5f1. (**F**) Quantitative analysis of wound repair capability of A549 LACSLCs containing siRNA-control or siRNA-MMP-2 at 24 h after seeding. (**G**) Quantitative analysis of invasive ability of A549 LACSLCs containing siRNA-control or siRNA-MMP-2. All experiments were performed at least in triplicates and data are presented as the mean ± SEM. Student *t* test was performed to evaluate the difference.

To confirm the inhibitory effect of POU5F1 downregulation on cell invasion and migration, we examined whether knockdown of POU5F1 could reduce the migratory and invasive potentials of LACSLCs. As shown in Figure 3, LACSLCs containing shRNA-Pou5f1 migrated much slower than control cells (Figure 3A and B). A549 LACSLCs with POU5F1 knockdown resulted in significantly decreased number of invading cells through the Matrigel to the lower chamber compared with shRNA-Control-infected A549 LACSLCs (Figure 3C and D). Furthermore, the tumors formed by A549 LACSLCs with POU5F1 knockdown were much smaller than control cells (Figure 3E), and displayed diminished invasion into muscle layers. These results suggest that higher migratory and invasive capabilities of LACSLCs result from the aberrantly high expression of POU5F1. Moreover, we found that the cell invasion and migration capabilities were significantly inhibited after MMP-2 knockdown in LACSLCs (Figure 3F and G).

### POU5F1 knockdown represses the expression and activity of MMP-2

**Figure 4:**
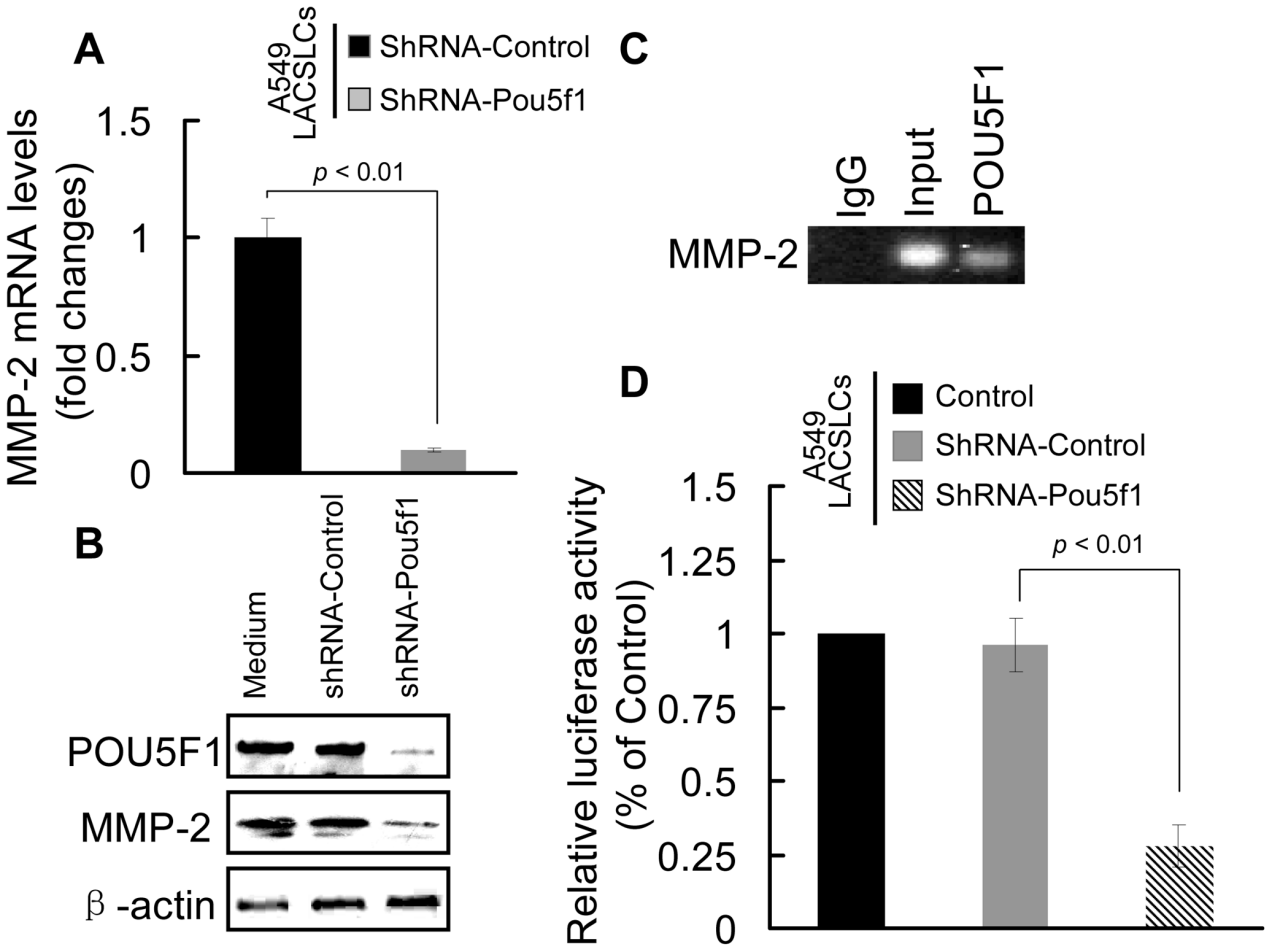
POU5F1 knockdown in A549 LACSLCs represses MMP-2 expression and activity. (A) qRT-PCR analysis of MMP-2 mRNA expression in A549 LACSLCs after POU5F1 knockdown. (B) POU5F1 knockdown decreases the protein expression of MMP-2 in A549 LACSLCs detected by western blot analysis. (C) ChIP analysis of transcript factor POU5F1 binding to MMP-2 promoter. (D) Luciferase reporter assay demonstrates inactivation of the MMP-2 promoter after POU5F1 knockdown in A549 LACSLCs. Cells were transiently transfected with a luciferase reporter construct using Lipofectamine 2000 reagent. After incubation for 24h, cells were harvested in passive lysis buffer, and the luciferase activities were measured by a luminometer using the luciferase assay system. All experiments were performed at least in triplicates and data are presented as the mean ± SEM. Student *t* test was performed to evaluate the difference.

Since POU5F1 and MMP-2 are significantly upregulated in LACSLCs, it would be of interest to investigate the relationship between these two molecules involved in cell invasion and migration capabilities. As shown in Figure 4A and B, qRT-PCR and western blot analysis revealed that the mRNA and protein levels of MMP-2 were markedly reduced in A549 LACSLCs containing shRNA-Pou5f1. ChIP analysis revealed that POU5F1 bound to the MMP-2 promoter, suggesting that POU5F1 might be responsible for transcriptional activation of MMP-2 (Figure 4C). Moreover, knockdown of POU5F1 significantly reduced the luciferase activity of a reporter construct containing MMP-2 promoter (Figure 4D). Our results demonstrate that POU5F1 in LACSLCs regulates MMP-2 expression by directly targeting MMP-2 transcription.

### POU5F1 and MMP-2 are correlated with the survival of LAC patients

**Figure 5:**
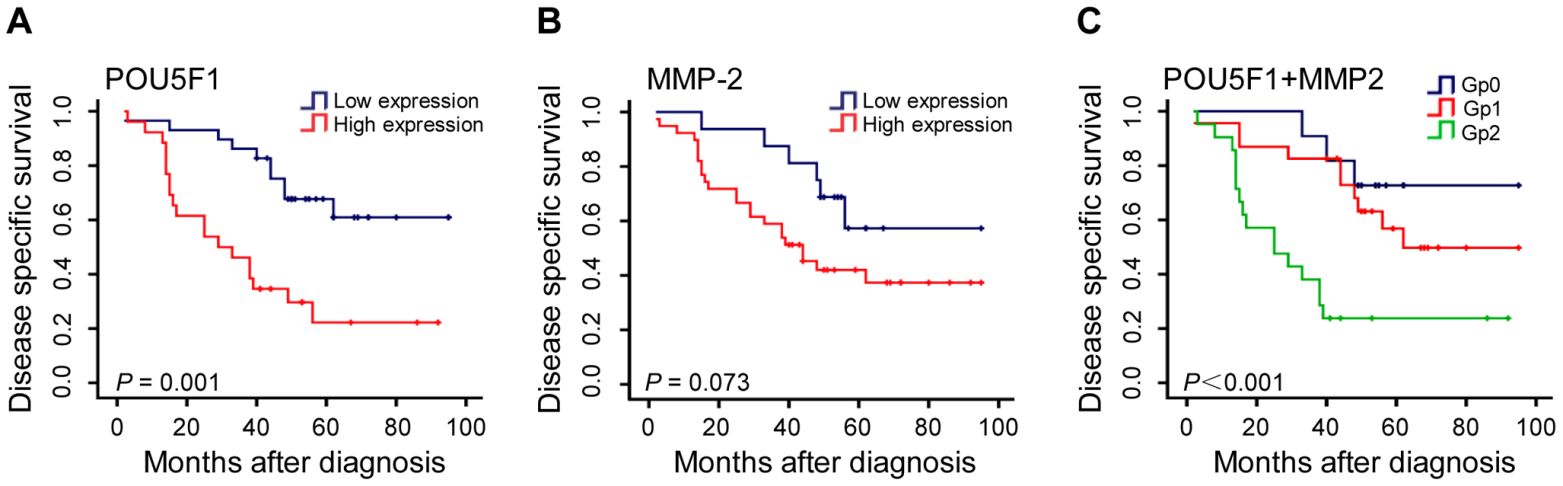
Characteristics of POU5F1 and MMP-2 expression in clinical tumor specimens and their correlations with LAC patients’ survival. (A) and (B) Kaplan–Meier analysis of the correlations of POU5F1 and MMP-2 with poor disease specific survival (DSS) in 55 LAC patients. (C) The combined expression of POU5F1 and MMP-2 indicates poor DSS for LAC patients. Double positive expression for POU5F1 and MMP-2 shows the worse prediction for the patient’s poor survival rates (*P*<0.001). Gp0: POU5F1 (low expression) and MMP-2 (low expression); Gp1: POU5F1 (high expression) or MMP-2 (high expression); Gp2: POU5F1 (high expression) and MMP-2 (high expression).

The clinicopathological correlations with the expressions of both POU5F1 and MMP2 were examined on a TMA containing 55 lung adenocarcinoma specimens to elucidate their associations with the survival of LAC patients (Table 1). Using the criteria described before, high expressions of POU5F1 (Figure 5A) and MMP-2 (Figure 5B) were observed in 26 (47.3%) and 39 (70.9%) of 55 lung adenocarcinoma specimens respectively. We found that the high expression of POU5F1 correlated with an ascending pathologic node (pN) stage for lung adenocarcinoma (*P* = 0.042).

By univariate analysis, high expressions of POU5F1 and MMP-2 were correlated with poor disease specific survival (DSS) of patients (*P* = 0.001, *P* = 0.073), respectively. Furthermore, we stratified patients into the three subgroups according to abovementioned two unfavorable factors, *i.e.,* Gp0, Gp1 and Gp2. As shown in Figure 5C, the median survival was the longest one in Gp0 versus the shortest one in Gp2 (i.e., 80.09 months for Gp0, 66.8 months for Gp1, and 38.1 months for Gp2, respectively, (*P*<0.001) (Figure 5C). Kaplan-Meier analysis demonstrated a significant impact of prognostic parameter tumor stage (*P* = 0.013) on DSS.

## Discussion

CSLCs have previously been reported in various cancers and proved to play critical roles in the cancer initiation, development and recurrence as well as cancer therapeutic failures [21]–[24]. To date, little is known regarding the invasive and migratory phenotypes and their regulatory mechanism of CSLCs. In this study, we found that transcription factor POU5F1 was required for LACSLCs invasion and migration, and it could enhance MMP-2 expression by directly targeting MMP-2 promoter to initiate transcription. Moreover, aberrantly high expressions of both POU5F1 and MMP-2 are correlated with the survival of patients with LAC.

It is well known that the POU domain transcription factor POU5F1 plays a pivotal role in the regulation of pluripotency characteristics in both somatic stem cells and CSLCs [8], [25]. Recently, it has been reported that POU5F1 induces CSLCs properties and contributes to the tumorigenesis and metastasis in LAC [9]. However, the mechanisms underlying the invasive and migratory potentials of CSLCs remain to be elucidated. Here, we confirmed that LACSLCs derived from A549 cells possess highly invasive and migratory abilities, and these cells expressed high levels of POU5F1 and MMP-2. MMP-2 and other members of the matrix metalloproteinases family are known to mediate invasion during development and metastasis, and are also regarded as putative tumor markers for clinical applications. [26]–[28]. MMP-2 degrades extracellular matrix and releases stored pro-migratory factors to allow movement of the cells within tissues [29]. However, MMP inhibitors have proved to be unsatisfactory in clinical trials due to either lack of selectivity towards MMPs or appearance of major adverse effects [16], [30]. The target-specific gene silencing of MMP-2 expression study exhibits the efficacy of siRNA-MMP-2 in inhibiting LACSLCs invasion and migration, suggesting that MMP-2 may act as a potent adjuvant for gene-targeted therapy in LAC.

The high expression levels of MMPs have been observed in the bladder cancer cells overexpressing POU5F1, suggesting that overexpression of POU5F1 upregulates genes of MMPs [11]. Our data indicate that both POU5F1 and MMP-2 may contribute to tumor initiation and invasion–metastasis phenotype of lung cancer. We revealed that the protein expression of MMP-2 was significantly decreased in LACSLCs after POU5F1 knockdown by shRNA strategy. Furthermore, ChIP and luciferase assays showed for the first time that depletion of POU5F1 with shRNA caused transcriptional downregulation of the endogenous MMP-2 gene and a MMP-2 promoter-reporter, suggesting that MMP-2 is a direct transcriptional target of POU5F1 in LACSLCs. Our findings support the concept that the necessary and sufficient action of POU5F1 in directly activating expression of specific target genes for promoting LACSLCs invasion and migration. There is also possibility that POU5F1 can repress certain genes, particularly in cases where it may bind cooperatively with other transcription factors with repression activity to enhance cell invasion and migration, and this might explain its association with corepressor complexes.

Since the pathogenesis and biologic behaviors of different subtypes of lung cancer are quite distinct, it is urgent to explore the advanced diagnostic methods and novel prognosis markers for different subtypes of lung cancer to improve the efficacy of cancer therapy. Our findings that POU5F1 can positively regulate MMP-2 expression to promote tumor cell invasion and migration of LACSLCs have important clinical implications. Although we did not observe a positive expression linkage between POU5F1 and MMP-2, the higher expression of each molecule was significantly associated with the poor prognosis, which is consistent with previous studies that POU5F1 and MMP-2 were reported to become independent risk factors for poor prognosis in lung cancer [14], [31]–[33]. More importantly, we here determined for the first time that combined overexpression of these two molecules presented the worst poor prognosis of LAC patients, suggesting that the expression of POU5F1 and MMP-2 provides an advantage for cancer cells to maintain invasion and migration property in LAC. Thus, the combined pathological examination of POU5F1 and MMP-2 by IHC can be used as an additional tool in identifying those LACs with high-levels of CSLCs to provide a strong predictive biomarker in clinical management of LAC patients.

In conclusion, our study demonstrates that POU5F1 and MMP-2 contribute to tumor invasion and migration phenotype of LACSLCs, and aberrantly high expression of POU5F1 can enhance MMP-2 expression by directly targeting the promoter of MMP-2. Moreover, we reveal that combined overexpression of POU5F1 and MMP-2 is correlated with the poor survival of patients with LAC. Our findings indicate that POU5F1 is required for controlling cell invasion and migration via direct regulation of MMP-2 in lung cancer, and support the idea that POU5F1 and MMP-2 will be useful as potential biomarkers of prognosis and novel targets for lung caner therapy.

## References

[1] Sutherland KD, Berns A (2010) Cell of origin of lung cancer. Mol Oncol 4: 397–403. 20594926 10.1016/j.molonc.2010.05.002 PMC5527931

[2] Eramo A, Lotti F, Sette G, Pilozzi E, Biffoni M, et al (2008) Identification and expansion of the tumorigenic lung cancer stem cell population. Cell Death Differ 15: 504–514. 18049477 10.1038/sj.cdd.4402283

[3] Sullivan JP, Minna JD (2010) Tumor oncogenotypes and lung cancer stem cell identity. Cell Stem Cell 7: 2–4. 20621039 10.1016/j.stem.2010.06.005 PMC3374716

[4] Levina V, Marrangoni A, Wang T, Parikh S, Su Y, et al (2010) Elimination of human lung cancer stem cells through targeting of the stem cell factor-c-kit autocrine signaling loop. Cancer Res 70: 338–346. 20028869 10.1158/0008-5472.CAN-09-1102 PMC4572892

[5] Rivera C, Rivera S, Loriot Y, Vozenin MC, Deutsch E (2011) Lung cancer stem cell: new insights on experimental models and preclinical data. J Oncol 2011: 549181. 21209720 10.1155/2011/549181 PMC3010697

[6] Duan JJ, Qiu W, Xu SL, Wang B, Ye XZ, et al (2013) Strategies for Isolating and Enriching Cancer Stem Cells: Well Begun Is Half Done. Stem Cells and Development 22: 2221–2239. 23540661 10.1089/scd.2012.0613 PMC3730373

[7] Xu C, Xie D, Yu SC, Yang XJ, He LR, et al (2013) beta-Catenin/POU5F1/SOX2 Transcription Factor Complex Mediates IGF-I Receptor Signaling and Predicts Poor Prognosis in Lung Adenocarcinoma. Cancer Res 73: 3181–3189. 23539445 10.1158/0008-5472.CAN-12-4403

[8] Chen YC, Hsu HS, Chen YW, Tsai TH, How CK, et al (2008) Oct-4 expression maintained cancer stem-like properties in lung cancer-derived CD133-positive cells. PLoS One 3: e2637. 18612434 10.1371/journal.pone.0002637 PMC2440807

[9] Chiou SH, Wang ML, Chou YT, Chen CJ, Hong CF, et al (2010) Coexpression of Oct4 and Nanog enhances malignancy in lung adenocarcinoma by inducing cancer stem cell-like properties and epithelial-mesenchymal transdifferentiation. Cancer Res 70: 10433–10444. 21159654 10.1158/0008-5472.CAN-10-2638

[10] Jung M, Peterson H, Chavez L, Kahlem P, Lehrach H, et al (2010) A data integration approach to mapping OCT4 gene regulatory networks operative in embryonic stem cells and embryonal carcinoma cells. PLoS One 5: e10709. 20505756 10.1371/journal.pone.0010709 PMC2873957

[11] Chang CC, Shieh GS, Wu P, Lin CC, Shiau AL, et al (2008) Oct-3/4 expression reflects tumor progression and regulates motility of bladder cancer cells. Cancer Res 68: 6281–6291. 18676852 10.1158/0008-5472.CAN-08-0094

[12] Kobayashi K, Takahashi H, Inoue A, Harada H, Toshimori S, et al (2012) Oct-3/4 promotes migration and invasion of glioblastoma cells. J Cell Biochem 113: 508–517. 21938739 10.1002/jcb.23374

[13] Bruna A, Darken RS, Rojo F, Ocana A, Penuelas S, et al (2007) High TGFbeta-Smad activity confers poor prognosis in glioma patients and promotes cell proliferation depending on the methylation of the PDGF-B gene. Cancer Cell 11: 147–160. 17292826 10.1016/j.ccr.2006.11.023

[14] Wang J, Pursell NW, Samson ME, Atoyan R, Ma AW, et al (2013) Potential Advantages of CUDC-101, a Multi-Targeted HDAC, EGFR, and HER2 Inhibitor, in Treating Drug Resistance and Preventing Cancer Cell Migration and Invasion. Mol Cancer Ther 12: 925–936. 23536719 10.1158/1535-7163.MCT-12-1045

[15] Yu SC, Xiao HL, Jiang XF, Wang QL, Li Y, et al (2012) Connexin 43 reverses malignant phenotypes of glioma stem cells by modulating E-cadherin. Stem Cells 30: 108–120. 22131169 10.1002/stem.1685

[16] Badiga AV, Chetty C, Kesanakurti D, Are D, Gujrati M, et al (2011) MMP-2 siRNA inhibits radiation-enhanced invasiveness in glioma cells. PLoS One 6: e20614. 21698233 10.1371/journal.pone.0020614 PMC3116828

[17] Wang CK, Cao JG, Peng B, Gu YX, Zheng GP, et al (2010) Inhibition of growth and motility of human A549 lung carcinoma cells by a recombined vascular basement membrane derived peptide. Cancer Lett 292: 261–268. 20053497 10.1016/j.canlet.2009.12.010

[18] Kim Y, Jeoung D (2009) The cancer/testis antigen CAGE induces MMP-2 through the activation of NF-kappaB and AP-1. BMB Rep 42: 758–763. 19944019 10.5483/bmbrep.2009.42.11.758

[19] Sobin LH WC, editors (2002) TNM classification of malignant tumors, 6th edition. New York: John Wiley & Sons.

[20] He LR, Zhao HY, Li BK, Zhang LJ, Liu MZ, et al (2010) Overexpression of AIB1 negatively affects survival of surgically resected non-small-cell lung cancer patients. Ann Oncol 21: 1675–1681. 20064830 10.1093/annonc/mdp592

[21] Marsden CG, Wright MJ, Pochampally R, Rowan BG (2009) Breast tumor-initiating cells isolated from patient core biopsies for study of hormone action. Methods Mol Biol 590: 363–375. 19763516 10.1007/978-1-60327-378-7_23

[22] Nagrath S, Sequist LV, Maheswaran S, Bell DW, Irimia D, et al (2007) Isolation of rare circulating tumour cells in cancer patients by microchip technology. Nature 450: 1235–1239. 18097410 10.1038/nature06385 PMC3090667

[23] Sueblinvong V, Weiss DJ (2010) Stem cells and cell therapy approaches in lung biology and diseases. Transl Res 156: 188–205. 20801416 10.1016/j.trsl.2010.06.007 PMC4201367

[24] Xu X, Dong Z, Li Y, Yang Y, Yuan Z, et al (2013) The upregulation of signal transducer and activator of transcription 5-dependent microRNA-182 and microRNA-96 promotes ovarian cancer cell proliferation by targeting forkhead box O3 upon leptin stimulation. Int J Biochem Cell Biol 45: 536–545. 23262295 10.1016/j.biocel.2012.12.010

[25] Babaie Y, Herwig R, Greber B, Brink TC, Wruck W, et al (2007) Analysis of Oct4-dependent transcriptional networks regulating self-renewal and pluripotency in human embryonic stem cells. Stem Cells 25: 500–510. 17068183 10.1634/stemcells.2006-0426

[26] Bjorklund M, Koivunen E (2005) Gelatinase-mediated migration and invasion of cancer cells. Biochim Biophys Acta 1755: 37–69. 15907591 10.1016/j.bbcan.2005.03.001

[27] Littlepage LE, Sternlicht MD, Rougier N, Phillips J, Gallo E, et al (2010) Matrix metalloproteinases contribute distinct roles in neuroendocrine prostate carcinogenesis, metastasis, and angiogenesis progression. Cancer Res 70: 2224–2234. 20215503 10.1158/0008-5472.CAN-09-3515 PMC2840052

[28] Wu KC, Yang ST, Hsia TC, Yang JS, Chiou SM, et al (2012) Suppression of cell invasion and migration by propofol are involved in down-regulating matrix metalloproteinase-2 and p38 MAPK signaling in A549 human lung adenocarcinoma epithelial cells. Anticancer Res 32: 4833–4842. 23155249

[29] Ye XZ, Xu SL, Xin YH, Yu SC, Ping YF, et al (2012) Tumor-associated microglia/macrophages enhance the invasion of glioma stem-like cells via TGF-beta1 signaling pathway. J Immunol 189: 444–453. 22664874 10.4049/jimmunol.1103248

[30] Chien MH, Lin CW, Cheng CW, Wen YC, Yang SF (2013) Matrix metalloproteinase-2 as a target for head and neck cancer therapy. Expert Opin Ther Targets 17: 203–216. 23252422 10.1517/14728222.2013.740012

[31] Shi M, Yu B, Gao H, Mu J, Ji C (2013) Matrix metalloproteinase 2 overexpression and prognosis in colorectal cancer: a meta-analysis. Mol Biol Rep 40: 617–623. 23184003 10.1007/s11033-012-2100-3

[32] Zhang X, Han B, Huang J, Zheng B, Geng Q, et al (2010) Prognostic significance of OCT4 expression in adenocarcinoma of the lung. Jpn J Clin Oncol 40: 961–966. 20462980 10.1093/jjco/hyq066

[33] Qian Q, Wang Q, Zhan P, Peng L, Wei SZ, et al (2010) The role of matrix metalloproteinase 2 on the survival of patients with non-small cell lung cancer: a systematic review with meta-analysis. Cancer Invest 28: 661–669. 20394501 10.3109/07357901003735634

